# Diagnosis of disease affecting gait with a body acceleration-based model using reflected marker data for training and a wearable accelerometer for implementation

**DOI:** 10.1038/s41598-023-50727-8

**Published:** 2024-01-11

**Authors:** Mohammad Ali Takallou, Farahnaz Fallahtafti, Mahdi Hassan, Ali Al-Ramini, Basheer Qolomany, Iraklis Pipinos, Sara Myers, Fadi Alsaleem

**Affiliations:** 1 Architectural Engineering Department, University of Nebraska–Lincoln, Omaha, NE 68182 USA; 2 Department of Biomechanics, University of Nebraska at Omaha, Omaha, NE 6160 USA; 3 Department of Surgery and VA Research Service, VA Nebraska-Western Iowa Health Care System, Omaha, NE 68105 USA; 4 Mechanical Engineering Department, University of Nebraska-Lincoln, Lincoln, NE 68588 USA; 5 Cyber Systems Department, University of Nebraska at Kearney, Kearney, NE 68849 USA; 6 Department of Surgery, University of Nebraska Medical Center, Omaha, NE 68105 USA

**Keywords:** Cardiology, Diseases, Health care, Medical research, Signs and symptoms, Mathematics and computing

## Abstract

This paper demonstrates the value of a framework for processing data on body acceleration as a uniquely valuable tool for diagnosing diseases that affect gait early. As a case study, we used this model to identify individuals with peripheral artery disease (PAD) and distinguish them from those without PAD. The framework uses acceleration data extracted from anatomical reflective markers placed in different body locations to train the diagnostic models and a wearable accelerometer carried at the waist for validation. Reflective marker data have been used for decades in studies evaluating and monitoring human gait. They are widely available for many body parts but are obtained in specialized laboratories. On the other hand, wearable accelerometers enable diagnostics outside lab conditions. Models trained by raw marker data at the sacrum achieve an accuracy of 92% in distinguishing PAD patients from non-PAD controls. This accuracy drops to 28% when data from a wearable accelerometer at the waist validate the model. This model was enhanced by using features extracted from the acceleration rather than the raw acceleration, with the marker model accuracy only dropping from 86 to 60% when validated by the wearable accelerometer data.

## Introduction

The current approaches for diagnosing cardiovascular diseases are limited in identifying individuals at risk, with most patients diagnosed at the late stages of their disease. For example, peripheral artery disease (PAD) is a highly prevalent cardiovascular syndrome produced by atherosclerotic blockages in the arteries supplying the legs. It is estimated to affect approximately 8.5 million people in the US^1,2^. However, 40–60% of patients with PAD were undiagnosed in a primary care setting^3^. This is partly because the symptoms and signs of PAD are frequently confused for common symptoms of aging. Moreover, the ankle-brachial index (ABI), the standard first test for PAD diagnosis, is a specialized test that is expensive, time-consuming, and only available in appropriately equipped and staffed vascular laboratories^4,5^.

Patients with PAD have a higher risk of stroke, heart attack, and death. Thus, a delay in diagnosis increases a patient’s health risks and overall medical treatment costs. Several investigators have proposed machine learning-driven diagnostic models for PAD to overcome these limitations based on machine learning^6–9^. Blood samples and Doppler data^10^, clinical records^11^, walking distances^12^, and arterial pulse waveforms^13^ are examples of the data resources used to train such machine learning models. Some of these models have achieved adequate accuracy, but significant limitations still exist in the time, resources, and expertise required to develop these models. More specifically, these models may require any of the following: (1) the gathering of detailed medical records (time), (2) labs with expertise in proteomic work and interviews that are not part of the standard of care (resources), and (3) involvement of physicians or providers with advanced training needed to gather the information required to develop accurate models (experts).

The literature has demonstrated the potential of machine learning models in utilizing vision-based and instrumented treadmill gait analysis to classify gait dysfunctions in patients with Multiple Sclerosis and Parkinson’s disease^14,15^. A similar approach can be used for PAD diagnostics. For example, A recent approach for diagnosing intermittent claudication (leg pain with walking), the most common, early manifestation of PAD, is using gait analysis data in machine learning models. Gait analysis is an accurate method of evaluating the mechanisms underlying functional impairments, quantifying the efficacy of treatment, and tracking PAD progression^12,16,17^. Specifically, compared with healthy controls, patients with PAD walk slower, take shorter steps before and after the onset of leg pain, and overall spend more time in the double support phase of walking, thus extending the stance time^12,18–20^. In light of such consistent findings^16,18,19,21–24^, we theorized that gait data could be analyzed to identify patterns and train machine learning models to indicate whether an individual has PAD.

A recent study in our laboratory used gait features extracted from ankle, knee, and hip kinetics and kinematics data, including joint angles, torques and powers, and ground reaction forces, to train machine learning models to classify individuals as patients with PAD or non-PAD controls^5^. Results showed that machine learning and gait features could classify individuals with PAD with acceptable performance (Accuracy: 89%, and Matthew's Correlation Coefficient: 0.64). One significant limitation of this and other similar works is the requirement of using motion capture systems that are using high-speed cameras to collect gait data for training and actual implementation, which is expensive, time-consuming, and inaccessible in most clinical settings^5^.

In concert with advanced algorithms, the advancement of wearable devices, such as accelerometers worn at the waist or the wrist, has opened the potential of such devices to gather detailed gait parameters outside laboratory settings^25–27^. It is, therefore, possible that wearable devices could offer a low-cost and more convenient tool for diagnosing PAD. However, the availability of extensive accelerometer data for many patients with PAD is relatively low. Another issue with existing data is a lack of consistency due to problems such as unwanted sensor movement. These limitations highlight the importance of data sources when developing prediction models, as poor data may result in models with poor diagnostic accuracy, limiting the advantages and wide adoption of wearable devices compared to standard medical diagnostic methods.

To overcome these challenges, we present a framework for generating acceleration data from previously available data in the literature. The presented framework classifies PAD by capitalizing on the benefits of gait analysis (precision and accuracy) while employing the simplicity of wearable accelerator measurements.

The framework presents a method to extract acceleration data from the motion of a reflective marker mounted at a specific anatomical position while subjects are walking. Collecting motion data from reflective markers is commonly available in biomechanics literature for gait evaluation. Also, multiple markers can be placed to simultaneously collect data on different body parts for the same experiment. Thus, the presented method eliminates the need for conducting a massive human subject experiment using traditional wearable accelerometer devices mounted on multiple anatomical locations to collect such data.

**Figure 1:**
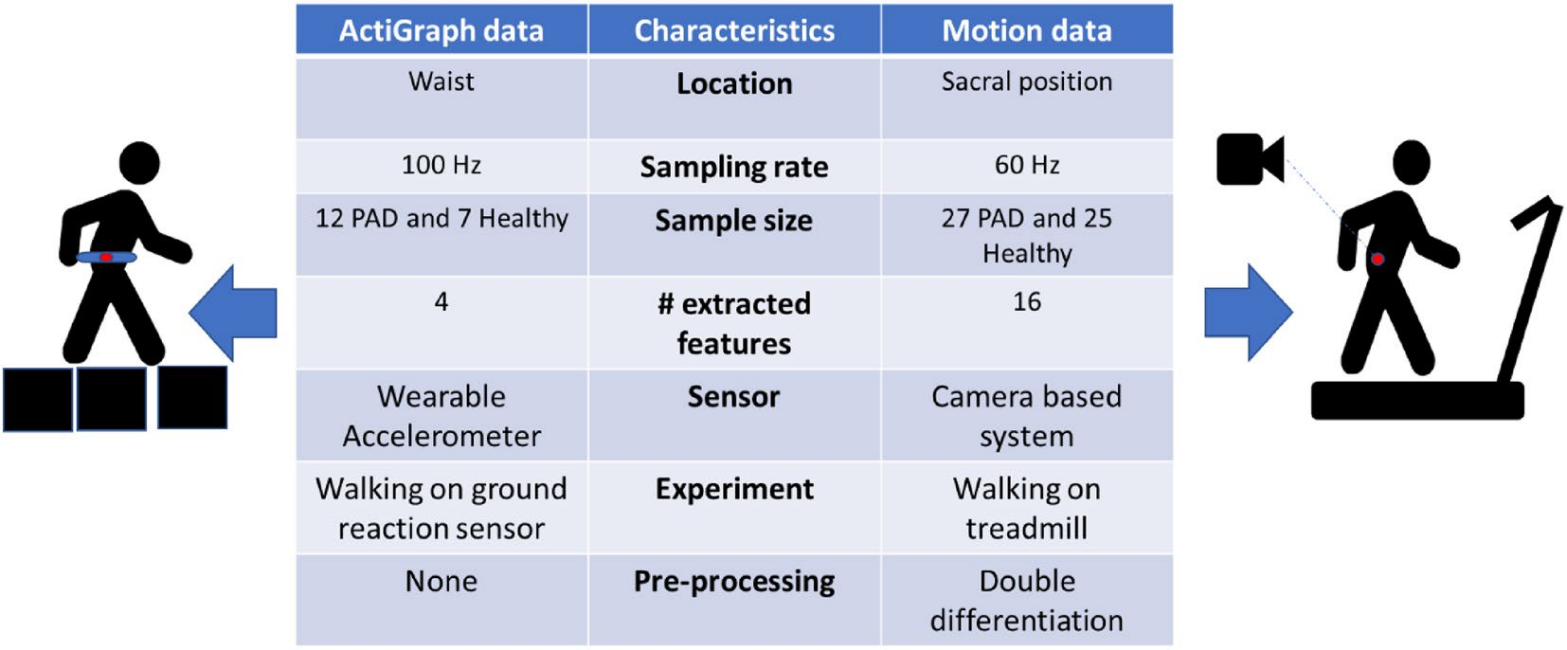
A summary of the two data sources used in this paper. The column on the left displays the characteristics of the time-series data obtained from the ActiGraph GT9X wearable accelerometer worn on the waist of the participants and sampled at 100 Hz. Meanwhile, the column on the right presents the traits of the marker data from different anatomical landmarks, including the sacrum, captured by infrared cameras and sampled at 60 Hz.

The ideal framework implementation will use already available anatomical marker data (precise and obtained under highly controlled conditions in a lab setting) to train the diagnostics model and then use wearable accelerometer measurements (enable diagnostics outside the lab environment) to test and apply this model. To evaluate such implementation, acceleration data were collected directly from a wearable ActiGraph GT9X accelerometer^28^ attached to the subject’s waist during overground walking. The waist position was chosen for the subject convenience. The two different data collection experiments are presented in Fig. 1 and were used to develop and validate the framework.

**Figure 2:**
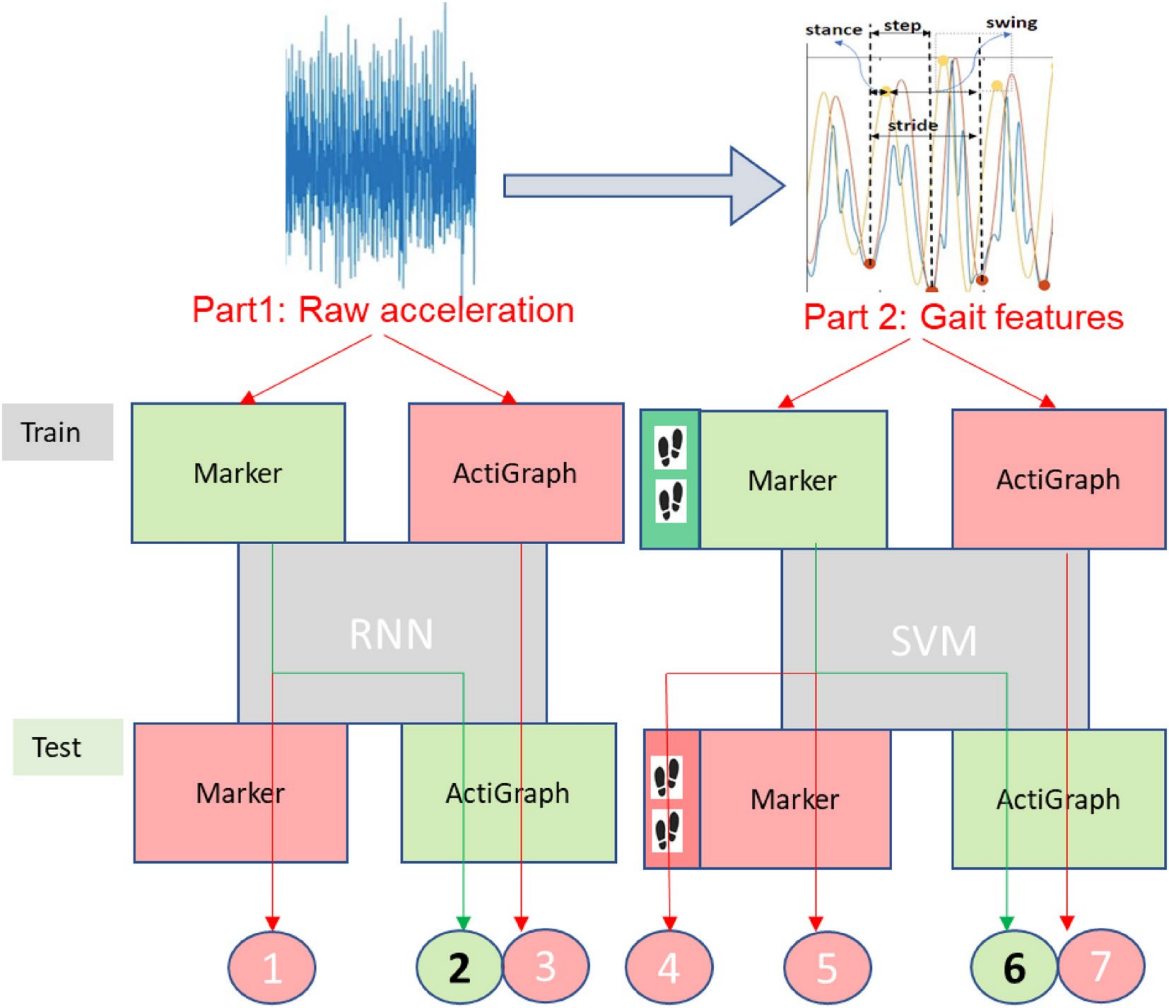
Flowchart showing our two approaches to establish the best diagnostics framework. The first deals with the raw acceleration data (Part 1), and the second deals with gait characteristics extracted from marker data (Part 2). A maximum of four (the primary) gait features were extracted from most of the data. Due to the high consistency among multiple gait cycles, the marker data at the sacrum position provided 16 gait features. The foot carton in Part 2 marks the extra gait features. Recurrent neural networks (RNNs) that used the acceleration time-series data as the LSTM model were used in part 1. Typical machine learning methods, such as the SVM that use the discrete extracted gait features, were used in part 2. In each part, different paths (labeled from 1 to 7) were used to find the best implementation of the framework. Each path has a training and testing module. Using the marker data in a test module or the raw acceleration in training, marked by red, represents a limitation.

For completeness, anatomical markers and ActiGraph data have been used in different combinations for training or testing. Each combination has distinct advantages and limitations (Fig. 2). For example, using marker data for training is advantageous, while its implementation in real-field settings has limitations^28^. On the other hand, the use and accessibility of the wearable accelerometer for real-field implementation is advantageous, but it is not as accurate for training models. As shown in Fig. 2, we also explored training recurrent neural network models (RNN) using the raw acceleration data versus standard machine learning models such as support vector machine (SVM) trained by their extracted features. The extracted features relate to gait characteristics such as stride, step, stance, and swing time. Sixteen features were extracted from the marker data at the sacrum position. However, only four features were derived from any other marker data point, including the ActiGraph acceleration data, due to consistency issues.

## Results

**Figure 3:**
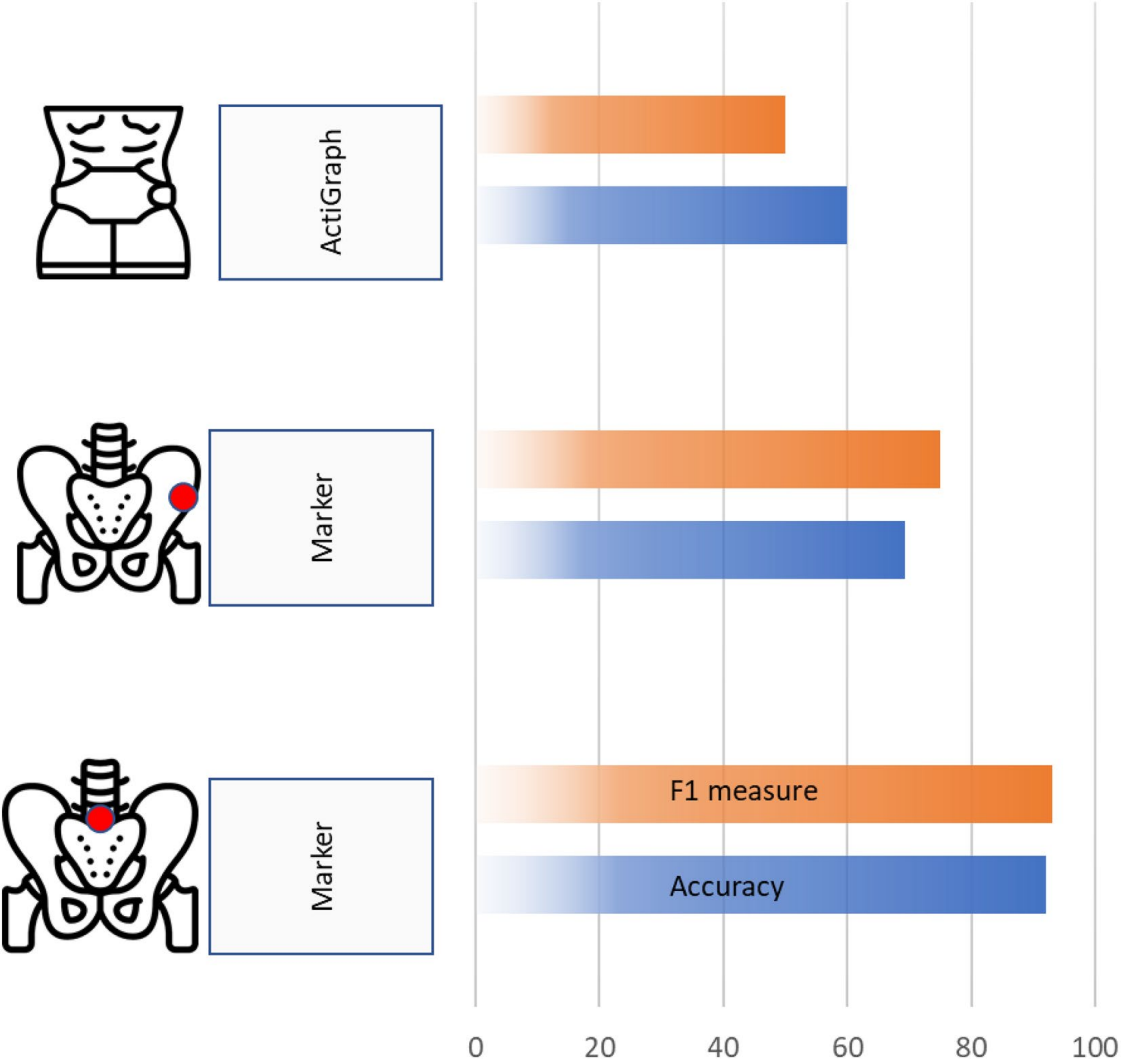
Different LSTM model performances were trained by raw acceleration data using data from markers mounted at the sacrum and the ASIS position and wearable accelerometers mounted near the waist. The F1 measure represents the precision and recall values.

Figure 3 compares the LSTM model performance using the raw acceleration data from the wearable accelerometer to those extracted from the sacral and anterior superior iliac spine (ASIS) marker. In this comparison, we adapt Paths 1 and 3 in Fig. 2 to eliminate the effect of using different data types in training and testing. Figure 3 shows that the models trained by the marker data at the sacrum (Path 1) perform better (accuracy of 92%) than those trained by the ASIS marker data (Path 1) and wearable accelerometer model (Path 3); due to symmetry, the sacral position has produced the most consistent data between gait cycles. Thus, the sacral marker data is used instead of the ASIS marker data in subsequent analysis.

Overall, results show clear advantages of using the extracted acceleration signals from marker data at the sacrum position to develop PAD diagnostics models. However, a wearable accelerometer is more feasible to implement. Thus, the best scenario is to use the marker-based extracted acceleration to build and train the diagnostics model and a wearable accelerometer for actual implementation. This scenario is represented by Path 2 in Fig. 2. In Path 2, we explore the potential of moving the marker-based model to an actual implementation. In this path, the wearable accelerometer data, while acknowledging the limitation it is not mounted at the sacrum position, test the model's accuracy trained by the sacral data. Unfortunately, the model produces a very low accuracy of 28%. In fact, the model predicted all subjects monitored by the wearable accelerometer to be healthy. We would expect better performance if the wearable accelerometer was mounted at the sacrum position. As we don’t have such data, next, we explore improving the model accuracy despite this limitation.

The low accuracy of the green Path 2 (Fig. 2) motivates the need to consider Part 2 (Fig. 2), which explores the potential of using extracted gait-related features rather than raw acceleration measurements in building the diagnostics models. Our hypothesis is using the features rather than the raw acceleration should reduce the model's dependency on the body part and thus produce better results even if the wearable accelerometer is not mounted at the sacrum position.

**Figure 4:**
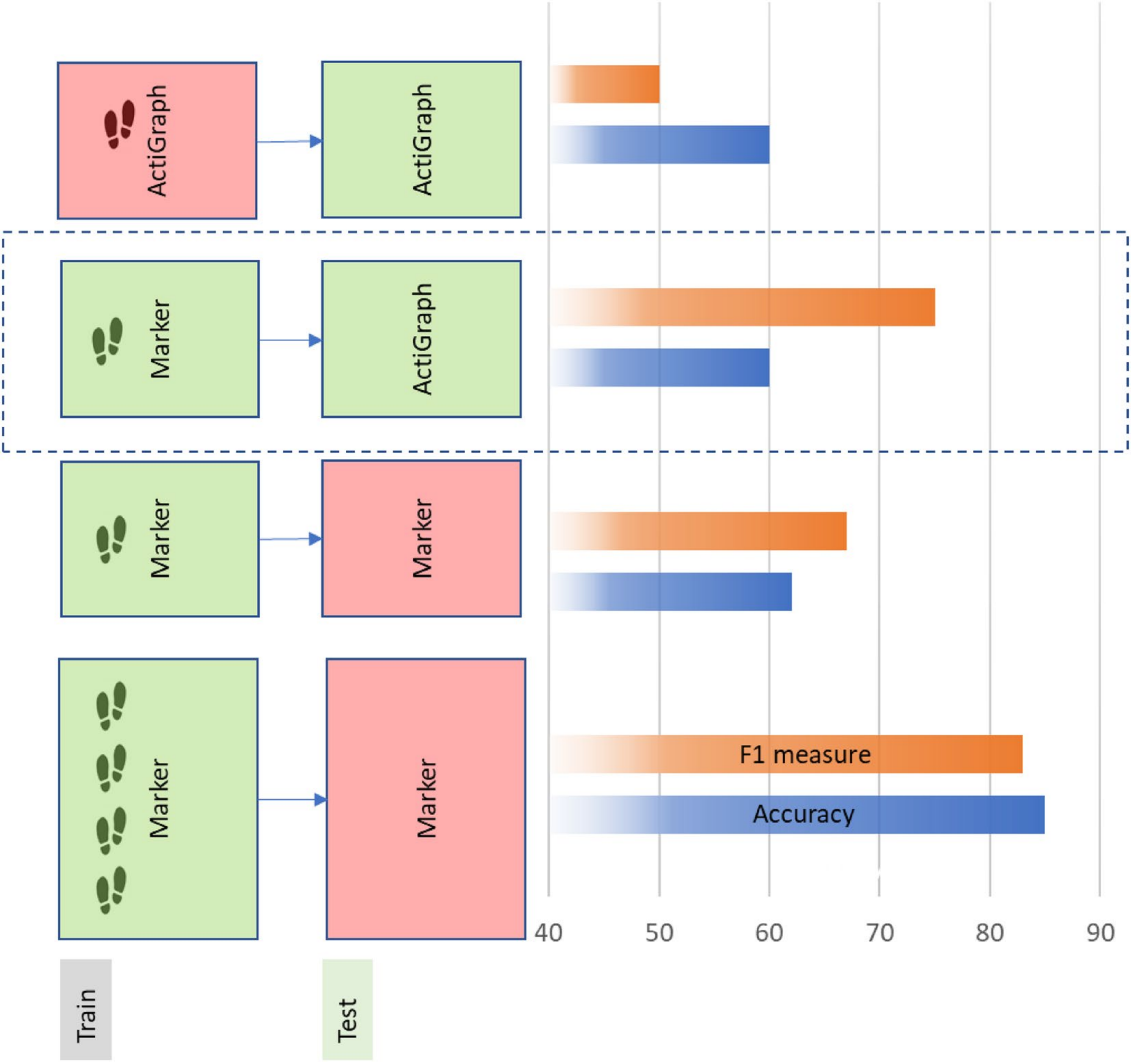
Different SVM model performances with different training and testing combinations of marker data mounted near the sacrum and wearable accelerometers near the waist. Up to 16 extracted gait-based features from the marker data are used in developing some of those models. But only 4 features were extracted from the wearable accelerometer due to consistency issues in the gait cycles.

Up to 85% accuracy is achieved using the SVM model (Path 4) when the 16 features extracted from the marker are used (Fig. 4). This accuracy drops to 62% when only the leading four features are used. An accuracy of only 60% was achieved when a model was trained and tested by the same four gait features extracted from the wearable accelerometer data. This observation validates the importance of measuring acceleration at the sacrum position. This translates to having multiple consistent walking steps (to generate the 16 features) to train machine learning.

Interestingly, as shown in Fig. 4, when tested by the wearable accelerometer data, the marker model trained by the four main gait features produced a similar accuracy of 60% and an even higher F1. This finding is significant as it represents the highest possible accuracy of the current data for the most practical implementation of the model, using the marker data for training and the wearable accelerometer for testing and validation. Although the marker position (sacral) differs from the wearable accelerometer position (waist), it matches the accuracy of the accelerometer data for training and testing. While this is still below the 85% accuracy when the model is trained and tested by the marker data, we expect the accuracy to get closer to 85% when the wearable accelerometer is mounted near the sacral position.

## Discussion

Our findings represent considerable progress in developing a model for PAD detection using gait features that can be captured in real-world settings. Previous work from our group classified individuals as having or not having PAD using biomechanics laboratory gait measurements and applying standard ML classifiers^5^. The datasets included detailed measurements of joint angles, torques, powers, and ground reaction forces. While this approach produces an accurate prediction of the presence of PAD in the subject tested, it is limited because of the requirement of performing the diagnostics in a biomechanics lab. To address this challenge, this paper presents the development of a framework that moves the application of this diagnostic model outside biomechanics labs. In establishing this framework, we used acceleration data collected using two methods. The first method involved wearing an ActiGraph GT9X accelerometer at the waist during normal overground walking. The other method involved measuring motion-derived acceleration from sacral markers during treadmill walking in the laboratory. We evaluated the raw acceleration data and also temporal gait features extracted from the raw acceleration data. Results indicate that using the temporal gait features improves the framework's performance and thus was used in this application.

Although the data came from different walking experiments and acceleration was collected at two different body parts, the model accuracy trained by the sacral marker data and evaluated by the ActiGraph wearable accelerometer data matches the accuracy of the model trained and evaluated by the accelerometer data.

The current standard of care for older individuals makes distinguishing between PAD symptoms and typical aging difficult, leading to many individuals not being diagnosed until PAD has progressed to advanced, limb-threatening stages^3^. Our work is a solid first step toward demonstrating the potential for developing classification and prediction models using temporal gait features that can be captured with wearable sensors in real-life settings. This method can be used in the patient’s own house and during regular everyday activities outside a clinical or research laboratory setting, as an initial indicator of the potential presence of PAD that would trigger standard vascular laboratory testing to confirm (or reject) the clinical diagnosis of PAD. It is possible that once the diagnosis is confirmed, the methodology can also be used to monitor disease progression and response to treatment, reflected in the deterioration or improvement, respectively, of the movement parameters. Based on these findings, it becomes evident that this application of wearable devices may directly impact clinical decision-making and could improve the quality of patient care.

Limitations of our work include: (1) The best results of the marker acceleration data were derived from the sacral position data. This position provides enough consistent walking steps to generate 16 gait features. On the other hand, the wearable accelerometer was only collected at the waist. Due to the limited consistent steps due to sensor movement and asymmetry, only 4 gait features were extracted at this body location. Better accuracy is expected when the wearable accelerometer is mounted at the sacral position. Future plans include recruiting new subjects with PAD and performing similar experiments while the wearable accelerometer is mounted near the sacrum to validate this hypothesis. (2) Data sets are small because it is challenging for patients with PAD to walk continuously for long distances due to the nature of their disease and symptoms. Future studies will ask patients with PAD to walk for multiple trials to get longer datasets and recruit more subjects to increase the overall amount of data.

## Methods

### System configuration and packages

This study analyzed, preprocessed, fine-tuned, and built the model on the University of Nebraska–Lincoln’s Holland Computing Center (HCC) Swan cluster, which has 56 cores and 256 GB of RAM per node. The cluster is powered by 168 Intel Xeon Gold 6348 CPUs, with 2 CPUs and 56 cores per node. The RAM configuration consists of 168 nodes, each with 256 GB and two nodes with 2000 GB of RAM. Each node offers 3.5 TB of local scratch storage, and approximately 5200 TB of shared Lustre storage is available.

We also used Python to be able to take advantage of HCC. We used Scipy for imputation and filtering, TensorFlow and Keras packages for model building, and Optuna and Hyperopt for tuning the machine-learning models.

### Participants

Patients with peripheral artery disease were recruited from the vascular clinics at the University of Nebraska Medical Center and the Omaha VA Medical Center. Subjects were aged 50 years and older, had a stable blood pressure, lipid, and diabetes regimen for 6 weeks, and positive history of chronic claudication, exercise-limiting claudication per history and direct observation, and evidence of occlusive disease on ankle/brachial index testing and/or computerized tomographic angiography. Subjects were excluded if walking capacity was limited by conditions affecting the legs (joint/musculoskeletal, neurologic) and systemic (heart, lung disease) pathology. Healthy older individuals had the same inclusion/exclusion criteria, except they had an ankle-brachial index above 0.90 and no history of claudication or exercise limitation as determined by a health history questionnaire. We did not include any patients without symptoms but with reduced blood flow (asymptomatic PAD).

### Data sources and preprocessing

Two different methods were used to obtain acceleration measurements. One method collected acceleration data directly from wearable accelerometers (ActiGraph GT9X Manufacturing Technology, Inc., FL, USA) attached to the subject's waist during overground walking conditions. The second method calculated acceleration data by double differentiating the position of a reflective marker mounted at the sacrum. Marker-position data is captured through a camera-based system while the subjects walk on a treadmill. The Internal Review Boards of the Nebraska Western Iowa Veteran Affairs and the University of Medical Centers approved both studies. All subjects provided informed consent before participation in the studies. The studies were conducted in accordance with the Declaration of Helsinki, and the Ethics Committee of IRB approved the protocol. Our data analysis for both experiments for diagnostic purposes is considered a secondary analysis.

The accelerometer data were collected from 12 patients with PAD and 7 healthy controls at a sampling rate of 100 Hz. The acceleration was captured in three dimensions (x, y, z). The x-axis denotes movement in the anterior–posterior, the y-axis represents movement in the mediolateral, and the z-axis represents movement in the vertical direction.

Motion capture marker position data was also collected in three dimensions (x, y, z) with a sampling rate of 60 Hz. Compared to the accelerometer data, the motion data were collected from 25 healthy individuals and 27 patients with PAD from flat treadmill walking trials. This led to an average of 30 steps per subject. The marker position data required the following steps to derive acceleration from the sacral marker trajectories^27,29–33^:

1. The difference between each pair of successive sample values (rows) was initially utilized to calculate displacement.
2. Velocity was calculated by dividing displacement by the time interval between every two consecutive points (1/60 s).
3. The difference between each consecutive sample velocity (rows) was used to calculate instantaneous velocity.
4. Acceleration was calculated by dividing instantaneous velocity by the time gap between each pair of adjacent points (1/60 s).
5. Noises were removed using the most popular method, a fourth-order Butterworth filter with a 15 Hz cutoff frequency.

**Figure 5:**
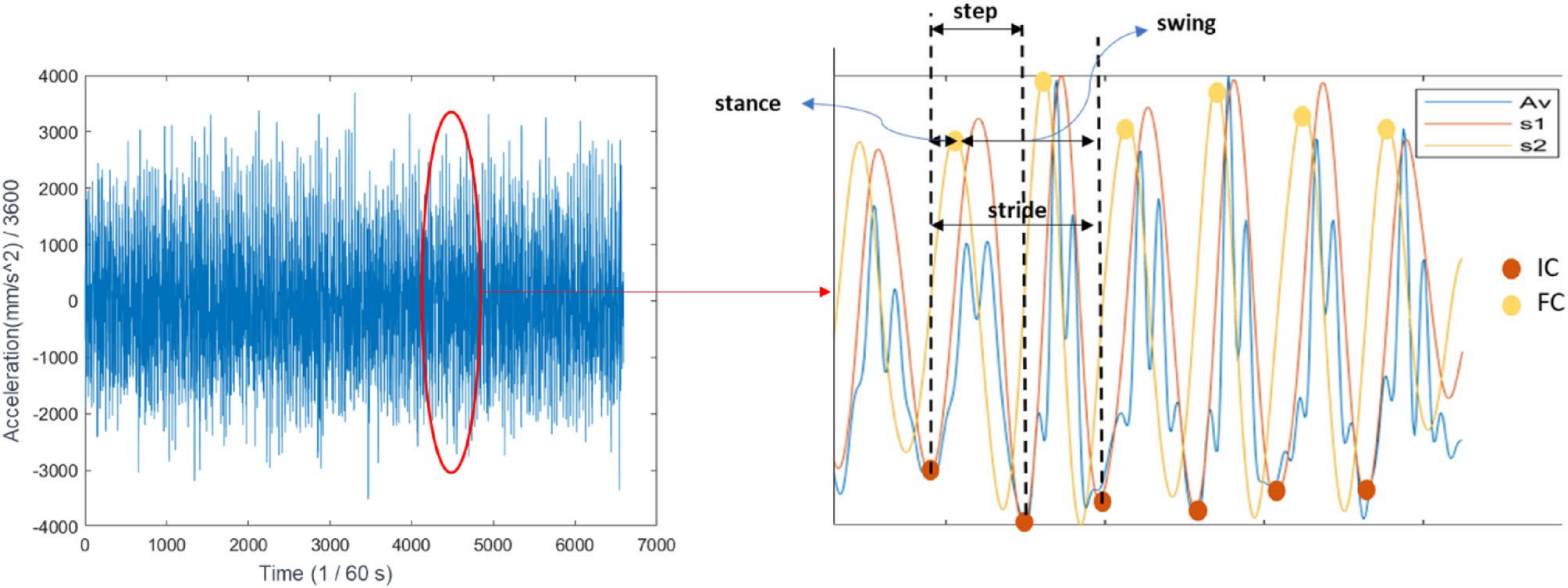
Graphic demonstration of gait cycles derived from the acceleration in the vertical direction obtained from the sacral marker trajectories (A_v_). S1 was derived from Gaussian Continuous Wavelet Transform (CWT) at a scale of 10 of vertical acceleration, and S_2_ was obtained from S_1_ differentiation. Initial contact was S_1_ local minima, and Final contact was S_2_ local maxima. Since we had consecutive steps, we assumed the first gait characteristics (like a step) as left (or right), then alternated values.

Once the acceleration measurements were available from both experiments, we followed steps to extract the gait characteristics (step, stance, stride, and swing time)^31,34^:

1. Identified the initial contact (IC, i.e. heel strike) and final contact (FC, i.e. toe-off) of a gait cycle using the wavelets temporal approach (Fig. 5). In this Figure, the following signals and points are shown:
  - A_v_ was the original acceleration signal.
  - S_1_ and S_2_ were the wavelet-transformed signals and its derivative, respectively.
  - IC and FC were defined as the minimum and maximum of S_1_ and S_2_, respectively.
2. Using the IC, FC, and gait cycles, extracted the following gait parameters:
  - step time: the interval between two Contralateral ICs,
  - stance time: time between heel strike (IC_i_) and toe-off (FC_i+1_),
  - stride time: the time between two ipsilateral ICs (IC_i+2_ and IC_i_),
  - swing time: the difference between stride and stance time.

Due to the many gait cycles for each subject in the acceleration data extracted from the motion, we extracted additional 12 gait characteristics. These characteristics are related to the variability of the four main step characteristics data and are defined as follows:

a- Magnitude of Variability (MV) = *σ (gait parameter),* where σ is the standard deviation,
b- Step Variability (SV) = $\sqrt{\frac{{Variance}_{left}+{Variance}_{right}}{2}}$, where variance_left_ and variance_right_ represent the variance of the gait characteristic for the left and right step, respectively.
c- Step Asymmetry (SA) $= \left|{average}_{left}-{average}_{right}\right|$, where the average left and average right represents the mean of the gait characteristic for the left and right legs, and asymmetry is the absolute mean difference between the right and left leg.

From the wearable accelerometer data, we identified the four temporal gait variables for machine learning: stride, step, stance, and swing time. However, we could not derive the remaining 12 gait characteristics from this data set, as they required many consecutive walking steps. Next, we explain in detail how these data will be used to train machine learning algorithms to classify individuals as having or not having PAD. We used the motion-based acceleration data as a case study in this explanation. Similar algorithms were followed when dealing with the accelerometer data while acknowledging that the data only produced four gait features compared to 16 features for the motion-based data.

### Models

Using the two sets of acceleration data, we applied two models to classify individuals as having or not having PAD. The first model utilized the recurrent neural network (RNN), which deals with time series data such as long short-term memory (LSTM)^35^, to enable using the raw acceleration measurements directly. In the second model, typical machine learning algorithms such as logistic regression, random forest, support vector machine, and deep neural network were trained with the gait characteristics extracted from the raw acceleration measurements. In contrast to the first model, the second model was designed with a simpler algorithm, while the complexity was shifted towards the input data. In other words, the second model relies more on the quality and quantity of the input data, while the first model may have a more complex algorithm to process the data.

## LSTM model using raw acceleration measurements

LSTM is an RNN with multiple layers of connected neurons. LSTM models include recurrent connections, which allow the state of the neuron from earlier activations in the preceding time step to be used as background for forming an output. To make a classification, input data is propagated through the network^36^. LSTM networks were explicitly designed to solve the RNNs issue with long-term dependencies. Because they have feedback connections, LSTM differs from more traditional feedforward neural networks (the second model approach in this study). This characteristic allows LSTM models to handle entire data sequences, like time series, without considering each data point in isolation. Instead, they can analyze current data by referring to preliminary data in the series^37–39^. Other types of RNNs, such as the standard RNN and the Gated Recurrent Units (GRUs) we also evaluated, are worth mentioning. However, they showed lower performance compared to the LSTM. Consequently, we narrowed our focus exclusively to the LSTM model for further analysis.

**Figure 6:**
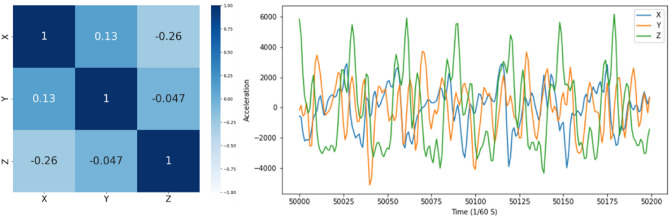
The left plot shows the correlation between the x, y, and z-axis of the motion-based acceleration data. A sample of three-dimensional acceleration can be seen in the right plot.

To train the LSTM model, the acceleration data were divided into training (two-thirds of the data including 15 healthy and 16 PAD subjects), testing (6 healthy and 7 PAD), and validation (4 healthy and 4 PAD) sets. Various ratios of PAD and healthy subjects in train, validation, and test sets were attempted before reaching the above combination, which resulted in the highest classification accuracy. Moreover, different combinations of the row acceleration time-dependent signals x, y, and z were used as input data for the LSTM model. The goal was to select the most informative dimensions of acceleration that enabled the LSTM model to classify faster and more accurately^40,41^. This was driven by the correlation study findings (Fig. 6), an example of the motion-based acceleration data showing the correlation between the acceleration axis measurements.

Next, we used acceleration calculated from sacral marker trajectory data while participants walked on the treadmill. The LSTM model achieved the best accuracy of 92% when it was trained using the y and z (mediolateral and vertical) accelerations (Table 1). Moreover, the x-axis acceleration measurement resulted in models with the highest accuracy if only one acceleration measurement was available (Table 1).

**Table 1:** All combinations of 3-axis time-series data fed to the LSTM model and the metrics obtained from predictions.

Combination	Accuracy (%)	Precision (%)	Recall (%)	F1 (%)
X, Y, Z	77	70	100	82
X, Y	62	62	71	66
X, Z	85	77	100	87
Y, Z	**92**	**88**	**100**	**93**
X	77	75	86	80
Y	54	54	100	70
Z	69	64	100	78

The best performance values are in bold.

We also evaluated the performance of different machine learning models to classify patients as having or not having PAD using the gait characteristics extracted from vertical acceleration (Fig. 4). For comparison purposes, we also showed the best performance of the LSTM model. The LSTM model still has the highest accuracy of 92% compared to 85% using the Logit or SVM model. It is worth mentioning the accuracy of the SVM model drops to 62% when only 4 features (the same features extracted from the accelerometer data) were used to train it. This observation validates the importance of having multiple consistent walking steps, that enable calculating additional gait variables for training machine learning models.

Finally, to harness the complete LSTM network’s capability, we tuned the hyperparameters (Table 2)^42^: number of steps (lookback size), maximum epochs, batch size, number of layers, number of neurons in each layer, activation functions, and optimizers. Our final LSTM model architecture and its tuned hyperparameters are an example of motion-based acceleration data models (Table 2). This architecture has an activation function before each hidden layer, two hidden layers, and a binary classification output activation function.

**Table 2:** The list of LSTM hyperparameters and the final values obtained from the grid search.

Hyper parameter	Description	Value
Number of steps	Number of observations in a single sequence of input data	100
Number of features	Number of input variables used to train the LSTM model	2
Max epochs	Maximum number of times the model iterates over the training dataset	1
Batch size	Number of samples processed by the model in each training iteration	4
Optimizer	Optimization algorithm used to minimize the loss function during training	Adam
Layers	The number of LSTM units stacked on top of each other	3 layers (4, 4, 1)
Activation function	Element-wise nonlinear function applied to the output of each LSTM unit	Tanh, sigmoid

## Standard machine learning models using the extracted gait characteristics

**Figure 7:**
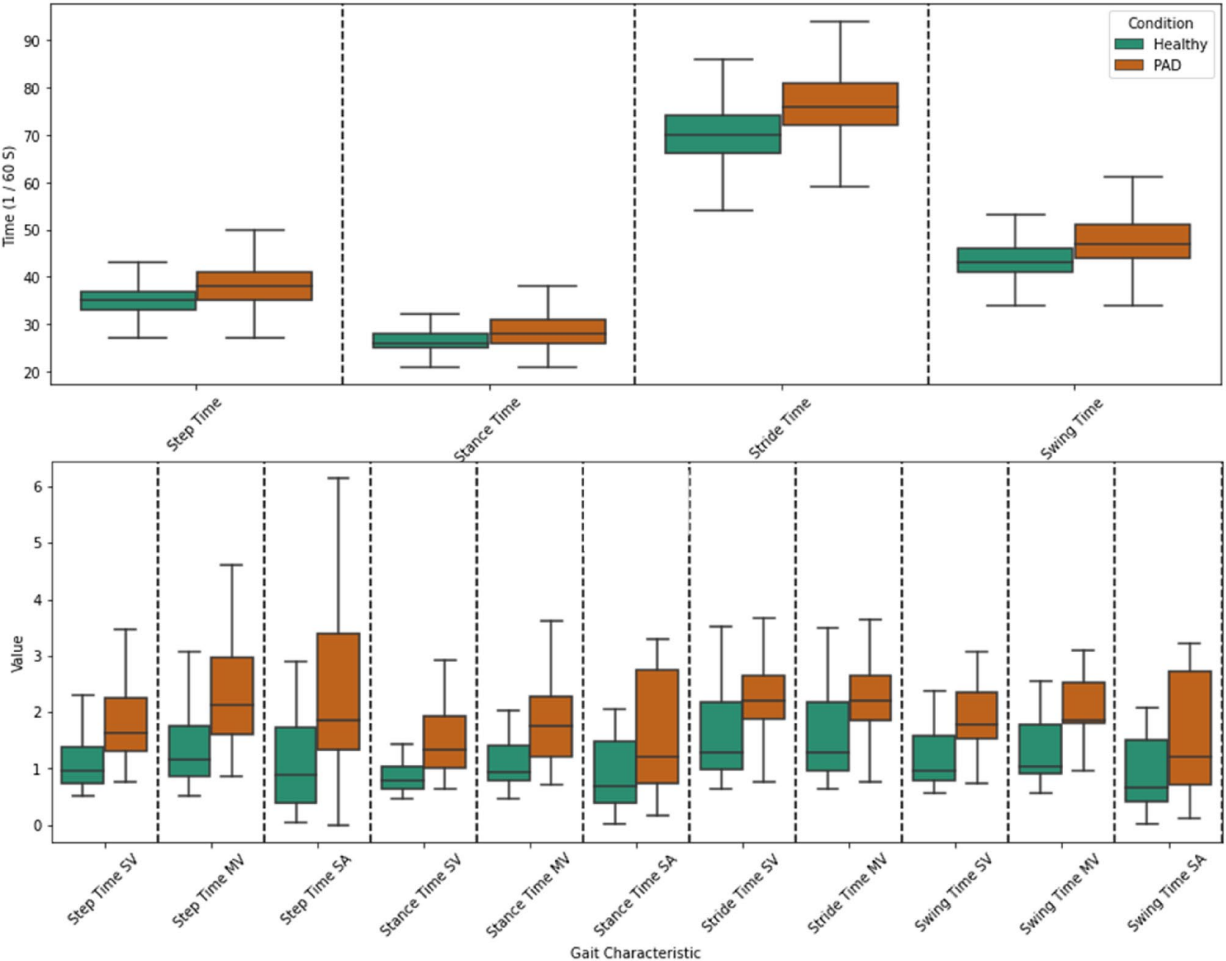
Box plot distributions of the extracted gait characteristics from the vertical acceleration of the motion-based data. The graphs demonstrate that gait characteristics have some distinguishable differences between patients with PAD and healthy controls. According to the box plots of the gait characteristics, there is a high level of separation between PAD and healthy controls in some variability and asymmetric features like 'SwingTime SV', 'SwingTime MV', 'StrideTime SV', and 'StepTime SA'.

Initial visualization of the extracted gait characteristics distribution revealed a distinguishable difference between PAD and healthy subjects (Fig. 7). The plots agreed with earlier research showing that, compared to healthy controls, PAD patients walk more slowly^12^. This finding motivated us to use the second modeling approach. In this approach, we only used the discrete values of the extracted temporal gait characteristics and ignored the acceleration time series data. This allowed us to use traditional (non-recurrent) machine learning algorithms such as logistic regression (Logit)^43,44^, random forest (RF)^45^, support vector machines (SVM)^46^, and deep neural networks (DNN)^47,48^ to classify individuals with PAD from healthy subjects. These algorithms are easier to train and implement than LSTM and have previously been employed in various classification tasks for other medical conditions^49,50^.

The gait data were separated into training and testing sets based on the subjects for the traditional machine learning models in a similar manner as described above for the LSTM approach. The machine learning models were tuned using a Bayesian algorithm and fivefold Cross-validation^51–53^. For missing values imputation, we grouped the gait data by subject and assigned the mean of the gait feature to the missing value^54^. Next, we present a detailed description of each model implementation.

### Logit model

We used the model summary and corresponding p-values of variables to identify the most important gait features. The initial model led us to remove four variables: "StepTime", "StanceTime", "StrideTime", and "SwingTime", as well as four variability features. We retained the remaining eight features ("StanceTime SV", "SwingTime SV", "StrideTime SV", "StepTime MV", "StepTime SA", "SwingTime SA", "StanceTime SA", and "StrideTime SA") to improve the performance of PAD/Healthy classification.

### RF model

**Figure 8:**
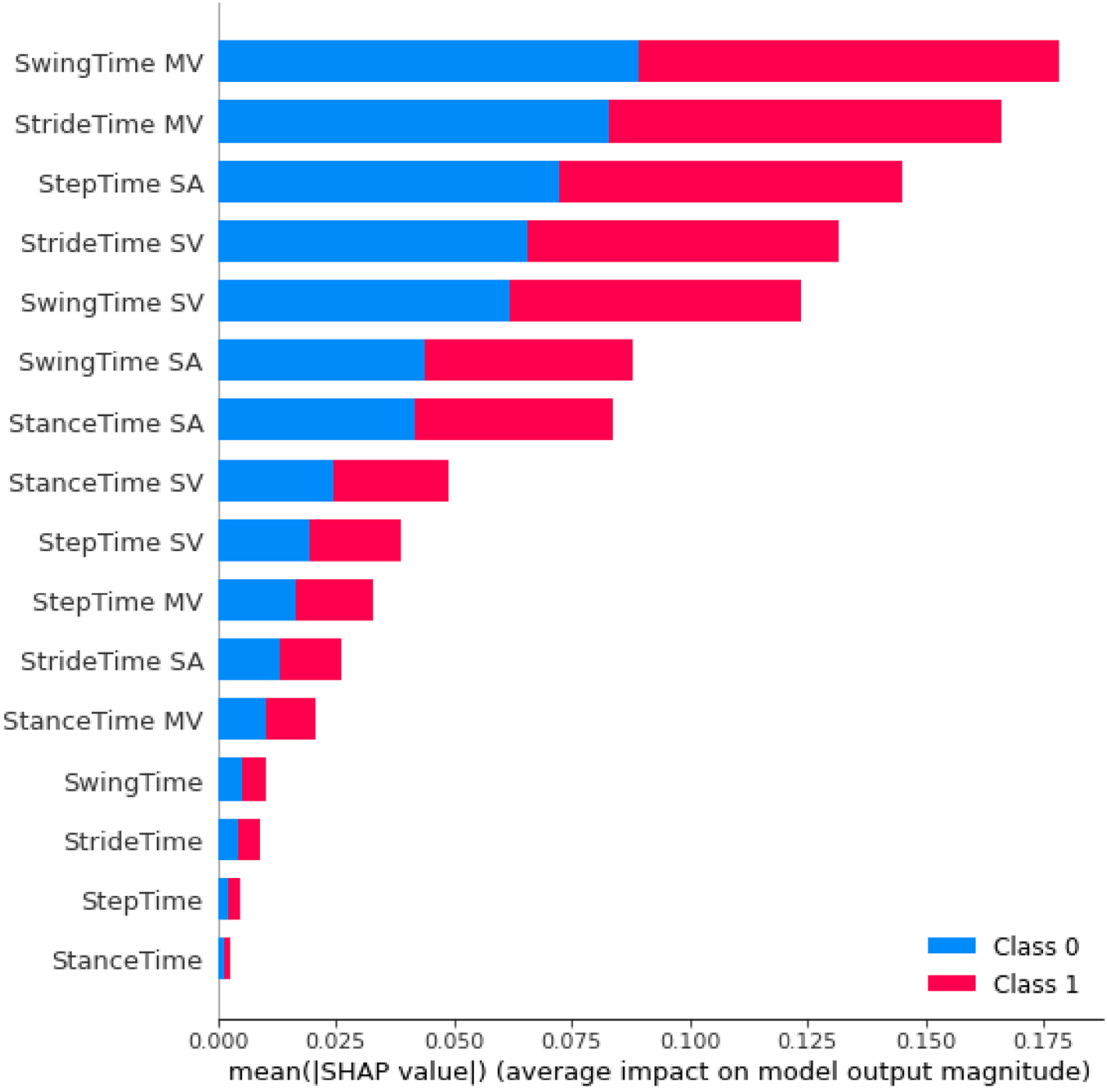
The SHAP summary plot for the Random Forest (RF) model was generated using all 16 features. The Y-axis represents the gait features, while the X-axis displays the mean absolute SHAP value. The plot shows the features sorted in descending order of importance, as determined by their mean absolute SHAP value. A SHAP value of 0 indicates that the feature does not impact the predicted output. A SHAP value of 0.2 indicates that the feature increases the predicted output by a moderate amount, while a SHAP value of 0.1 indicates that the feature increases the predicted output by a smaller amount.

The hyperparameters (Table 3) were tuned on the training set, and finally, the less significant features were found and removed based on their SHAP (SHapley Additive exPlanations) values (Fig. 8). The SHAP value has been presented as a way to quantify feature importance since the value it assigns to each feature reflects its role in model prediction^55,56^. At the end, the top 4 features, were used to train the final RF model.

**Table 3:** Hyperparameters used to tune the RF model, their range, and the result of tuning.

Hyperparameter	Description	Range	Selected
Criterion	Function used to measure the quality of a split in a decision tree	Entropy, gini	Entropy
max_depth	Maximum depth of each tree in the forest	Range (0, 20)	1
max_features	Number of features to consider when looking for the best split	Auto, sqrt, log2, None	log2
min_samples_leaf	Minimum number of samples required to be at a leaf node	Uniform (0, 0.3)	0.1585
min_samples_split	Minimum number of samples required to split an internal node	Uniform (0, 1)	0.106
n_estimators	Number of trees in the forest	Range (0, 100)	78

Based on the SHAP summary plot, we identified and kept the four most important features ("SwingTime MV", "SwingTime SV", "StrideTime SV", "StepTime SA") returned the hyperparameters and built the final Random Forest model.

### SVM

A similar approach to the RF model was used to find the hyperparameters for the SVM model (Table 4).

**Table 4:** Hyperparameters used to tune the SVM model, their range, and the result of tuning.

Hyperparameter	Description	Range	Selected
C	Controls the trade-off between allowing misclassifications on the training set and forcing the decision boundary to fit the data more closely	Range (0, 100)	65
Kernel	Specifies the type of kernel to use for the SVM	'Linear', 'poly', 'rbf', 'sigmoid'	'poly'
Degree	Specifies the degree of the polynomial kernel function	1, 2, 3, 4, 5	4
Probability	Specifies whether to enable probability estimates	Probability	0

### DNN

We used the Bayesian hyperparameter tuning approach for the DNN model, including the number of hidden layers, the number of neurons in each hidden layer, activation functions, optimizers, and maximum epochs. Based on our previous findings, we also dropped the following features: 'StepTime', 'StanceTime', 'StrideTime', and 'SwingTime'). The final list of hyperparameters of the DNN model is shown in Table 5.

**Table 5:** Hyperparameters used to tune the DNN model, their range, and the result of tuning.

Hyperparameter	Description	Range	Selected
Layers	Individual building blocks of a neural network	Range (2, 5)	4
Neurons	Basic computational units within a neural network	Arange (2, 256, 4)	64, 256, 64, 1
Dropout	Regularization technique commonly used in neural networks to prevent overfitting	Arange (0.20, 0.75, 0.025)	0.7
Batch size	How many training examples are processed in a single forward/backward pass during each training iteration	Arange (4, 128, 8)	44
Activation function	Neural networks can learn intricate relationships between inputs and outputs by adding non-linearities to a neuron's output	Relu, tanh, sigmoid	Sigmoid
Optimizer	Algorithm used to modify a neural network's weights and biases while it is being trained	Adadelta, adam, rmsprop, adagrad, sgd	Adam

To evaluate the different models in this study, we adopted the typical machine learning metrics such as accuracy, precision, recall, and F1 score. Accuracy is measured as the proportion of correctly classified instances to all instances. The degree of precision indicates how frequently the positive class label has been mistakenly assigned to another class. Recall gauges the accuracy of our model's True positive predictions. The balance between recall and precision is represented by the F1-score^57^.

In other words, precision indicates the probability that if the model classifies someone as a PAD patient, they actually have PAD. On the other hand, recall indicates the probability that if someone has PAD, the model can correctly identify them as having PAD. Thus, in addition to accuracy, F1 can be a considerable metric that reflects both precision and recall.

## Data Availability

The datasets used and/or analyzed during the current study available from the corresponding author on reasonable request.
